# Common carotid artery diameter and the risk of cardiovascular disease mortality: a prospective cohort study in northeast China

**DOI:** 10.1186/s12889-024-17749-x

**Published:** 2024-01-22

**Authors:** Ziyi Yin, Jiajing Guo, Ru Li, Hong Zhou, Xue Zhang, Shanshan Guan, Yuanmeng Tian, Li Jing, Qun Sun, Guangxiao Li, Liying Xing, Shuang Liu

**Affiliations:** 1 Department of Ultrasound, The Fourth Affiliated Hospital of China Medical University, Shenyang, 110000 People’s Republic of China; 2 Department of Ultrasound, The Affiliated Hospital of Northwest University, Xi’an No.3 Hospital, Xi’an, 710000 People’s Republic of China; 3 Department of Cardiovascular Ultrasound, Central Hospital of Chaoyang City, Chaoyang, 122000 People’s Republic of China; 4 Department of Chronic Disease, Liaoning Provincial Center for Disease Control and Prevention, Shenyang, 110005 People’s Republic of China; 5 Department of Chronic Disease, Disease Control and Prevention of Chao Yang City, Chaoyang, 122000 People’s Republic of China; 6 Department of Medical Record Management Center, The First Hospital of China Medical University, Shenyang, 110001 People’s Republic of China

**Keywords:** Common carotid artery, Cardiovascular disease, Mortality, Epidemiology

## Abstract

**Background:**

The association between the common carotid artery (CCA) diameter and cardiovascular disease (CVD) is recognized, but the precise nature of this link remains elusive. This study aimed to investigate the potential relationship between CCA diameter and the risk of CVD mortality in a large population in northeast China.

**Methods:**

The current study included 5668 participants (mean age 58.9 ± 10.1 years) from a population-based study conducted in rural areas of northeast China between September 2017 and May 2018. Information on death was collected from baseline until July 31, 2022. The CCA inter-adventitial diameter was measured using ultrasound. Cox proportional-hazard models were employed to explore the relationship between the common carotid artery diameter and cardiovascular mortality.

**Results:**

At baseline, the mean CCA diameter (mm) of subjects was 7.30 ± 0.99 and increased significantly with age, ranging from 6.65 ± 0.71 among people 40–49 years to 7.99 ± 1.04 among people ≥ 80 years. CCA diameter was significantly larger in males compared to females (7.51 ± 1.03 versus vs. 7.16 ± 0.94; *P* < 0.001). A total of 185 participants died of CVD during a median follow-up of 4.48 years. CCA diameters were divided into quartiles, and the highest quartile of carotid diameter (≥ 8.06 mm) had a 2.29 (95% confidence interval [CI]: 1.24, 4.22) times higher risk of CVD mortality than the lowest quartile (≤ 6.65 mm) (*P* < 0.01) in the fully adjusted model. Each increase in the diameter of the common carotid artery (per SD) raised the risk of cardiovascular death by 36% (hazard ratio [HR]: 1.36; 95% CI: 1.18, 1.57). The subgroup analysis results demonstrated that a per SD increase was associated with a 42% increased risk of CVD mortality in participants aged ≥ 64 years in the fully adjusted model (HR: 1.42; 95%CI: 1.21, 1.66).

**Conclusions:**

Our study indicates the possible incremental value of CCA diameter in optimizing the risk stratification of cardiovascular disease and provides essential insights into reducing the burden of cardiovascular disease.

## Introduction

Cardiovascular disease (CVD) persists as the foremost cause of premature mortality and disability, exerting a substantial toll on healthcare systems and societal productivity [1, 2]. Globally, CVD is responsible for roughly 33% of all deaths [3], with China exhibiting the highest cardiovascular disease mortality rates worldwide [4]. Consequently, a thorough comprehension of the risk stratification for CVD is imperative for enhancing preventive measures and mitigating adverse outcomes.

CCA diameter, measured on ultrasound images as the distance between adventitia interfaces, is believed to increase due to hypertension and may correlate with growing arterial wall thickness [5]. While numerous prior studies have concentrated on assessing the intima-media thickness (IMT) of the common carotid artery [6, 7], some investigations have highlighted that an increased common carotid diameter is independently linked to various cardiovascular risk factors. Moreover, it has been suggested that the common carotid diameter could offer more predictive value for all-cause mortality than IMT [8–10]. A comprehensive meta-analysis involving four cohort studies in Western countries has indicated that a larger carotid artery diameter may be associated with an increased risk of CVD mortality [11]. Unfortunately, few studies have explored the relationship between CCA diameter and CVD mortality within Asian populations, particularly in northeast China. Hence, it is imperative to undertake a study in northeastern China to unravel the connection between CCA enlargement and the risk of CVD mortality, potentially enhancing prognostic insights for individuals with CVD.

In this study, our objective was to assess the association between CCA enlargement and CVD mortality in a diverse general population in northeastern China through a prospective community-based cohort study. Additionally, we aimed to delve into the potential of CCA in optimizing CVD risk stratification in these specific regions.

## Methods

### Study population

**Fig. 1:**
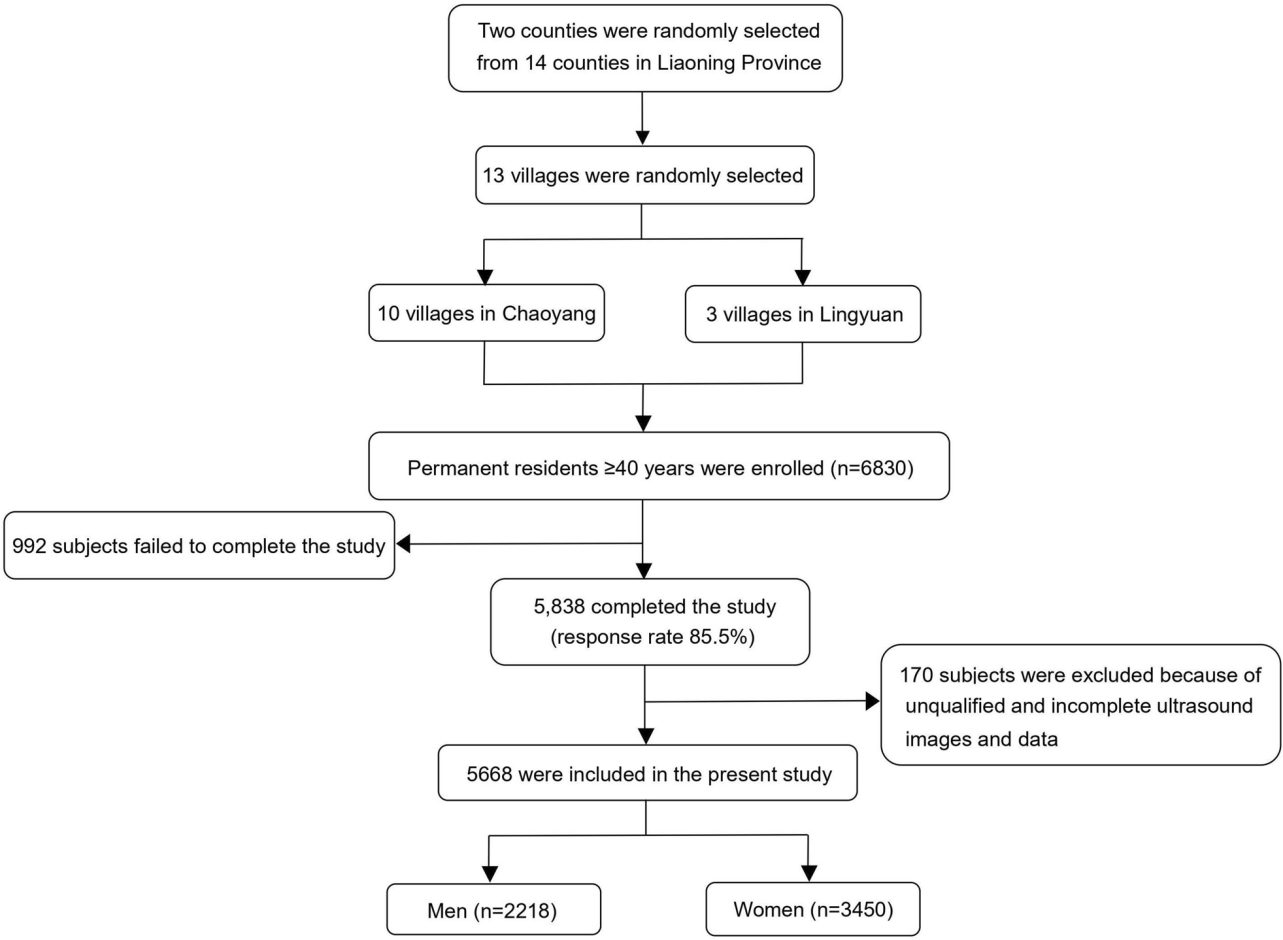
Flowchart of study population selection

This population-based prospective cohort study comprised individuals from two counties in northeast China, as illustrated in Fig. 1. A total of 6,830 participants aged 40 years and above were initially recruited from 13 villages in Liaoning Province between September 2017 and May 2018. However, only 5,838 participants completed the study during this period. Baseline carotid ultrasound images were obtained, and 170 subjects with ineligible images were subsequently excluded. Ultimately, 5668 participants were included in the study, and detailed characteristics are presented in Table 1. A follow-up period of approximately 5 years was conducted to monitor the survival or death status of these participants until July 31, 2022. The study adhered to the ethical standards outlined in the 1964 Declaration of Helsinki and received approval from the Central Ethics Committee of the China National Center for Cardiovascular Disease. All participants provided written informed consent.

**Table 1:** Characteristics of the study population

Characteristics	Total (*n* = 5668)	Carotid Diameter Quartiles, mm			
		Q1 (≤ 6.65, *n* = 1609)	Q2 (6.66–7.25, *n* = 1423)	Q3 (7.26–8.05, *n* = 1476)	Q4 (≥ 8.06, *n* = 1160)
Age (x), y	58.9 ± 10.1	52.9 ± 8.5	57.0 ± 9.0	61.7 ± 9.6	65.8 ± 8.5
Sex, n (%)					
Male	2218 (39.1)	469 (29.1)	522 (36.7)	633 (42.9)	594 (51.2)
Female	3450 (60.9)	1140 (70.9)	901 (63.3)	843 (57.1)	566 (48.8)
Education level, n (%)					
Primary school or lower	3256 (57.4)	748 (46.5)	807 (56.7)	900 (61.0)	801 (69.1)
Middle school	1783 (31.5)	631 (39.2)	457 (32.1)	420 (28.5)	275 (23.7)
High school or above	629 (11.1)	230 (14.3)	159 (11.2)	156 (10.6)	84 (7.2)
Income (CNY), n (%)					
< 5000	2041 (36.0)	410 (25.5)	467 (32.8)	593 (40.2)	571 (49.2)
5000–9999	1327 (23.4)	365 (22.7)	330 (23.2)	352 (23.8)	280 (24.1)
10,000–19,999	1146 (20.2)	385 (23.9)	310 (21.8)	266 (18.0)	185 (15.9)
>= 20,000	1154 (20.4)	449 (27.9)	316 (22.2)	265 (18.0)	124 (10.7)
Heart Rate, bpm	72.9 ± 12.8	71.5 ± 11.4	72.3 ± 12.1	74.0 ± 13.6	74.1 ± 14.2
Body mass index, kg/m^2^	24.7 ± 3.8	24.5 ± 3.7	25.0 ± 3.6	24.6 ± 3.7	24.8 ± 4.0
Current smoking, n (%)	1458 (25.7)	338 (21.0)	364 (25.6)	411 (27.8)	345 (29.7)
History of stroke, n (%)	349 (6.2)	40 (2.5)	84 (5.9)	118 (8.0)	107 (9.2)
History of CHD, n (%)	175 (3.1)	22 (1.4)	36 (2.5)	67 (4.5)	50 (4.3)
Atrial fibrillation, n (%)	61 (1.1)	8 (0.5)	11 (0.8)	16 (1.1)	26 (2.2)
Hypertension, n (%)	3245 (57.3)	600 (37.3)	780 (54.8)	966 (65.4)	899 (77.5)
SBP, mmHg	141.6 ± 22.8	131.2 ± 19.5	139.0 ± 21.0	146.0 ± 22.5	153.6 ± 22.6
DBP, mmHg	87.3 ± 11.8	83.9 ± 10.7	86.8 ± 11.2	88.8 ± 12.1	90.5 ± 12.2
Diabetes mellitus, n (%)	713 (12.6)	141 (8.8)	187 (13.1)	210 (14.2)	175 (15.1)
TC (mmol/L)	5.0 ± 1.0	4.9 ± 1.0	5.0 ± 1.1	5.0 ± 1.0	5.0 ± 1.0
TG (mmol/L)	1.5 ± 1.2	1.4 ± 0.9	1.5 ± 1.3	1.6 ± 1.3	1.5 ± 1.1
HDL-C (mmol/L)	2.3 ± 0.7	2.2 ± 0.67	2.3 ± 0.7	2.4 ± 0.7	2.4 ± 0.7
LDL-C (mmol/L)	2.0 ± 0.9	2.0 ± 0.9	2.0 ± 0.9	1.9 ± 0.9	2.0 ± 0.9

Continuous variables are expressed as mean ± standard deviation (SD) and categorical variables as number (%)

*CNY* Chinese yuan (1CNY = 0.15 USD), *CHD* Coronary heart disease, *SBP* Systolic blood pressure, *DBP* Diastolic blood pressure, *TC* Total cholesterol, *TG* Triglyceride, *HDL-C* High-density lipoprotein cholesterol, *LDL-C* Low-density lipoprotein cholesterol

### Demographic and clinical data

All participants completed a questionnaire covering demographic variables (such as age, sex, education, and income), smoking habits, and disease history (including stroke, coronary heart disease, atrial fibrillation, hypertension, and diabetes) during baseline interviews. Clinical examinations included measurements of heart rate, body mass index, blood lipid levels, and blood pressure levels. Previously established data collection methods and definitions were employed, including criteria for current smoking, hypertension, and diabetes.

### Carotid ultrasonography

Participants underwent carotid duplex ultrasonography using a high-resolution ultrasonography device (Mindray M7, Shenzhen, China) equipped with a broadband 7L4S linear array transducer. The measurements were conducted by highly trained and certified sonographers with over 3 years of experience in vascular ultrasound imaging. Rigorous training of team members was conducted before data collection. Subjects were examined in a supine position, and both sides of the carotid arteries were scanned.

At diastole, CCA diameter between the adventitia interfaces was imaged along the long axis of the vessel (1–1.5 cm segment proximal to the dilation of the carotid bulb). The diameter value was recorded as the average value of the left and right carotid, and the mean value of both sides was used for statistical analysis. Intra-reader reliability was assessed in 50 randomly selected subjects from all participants. CCA diameters were examined twice in a blind manner with an interval of 1 week.

### Ascertainment of outcomes

We obtained information about death, including the date and cause of death, from the National Population Registry of the China National Statistical Office. Mortality outcomes were assessed during the follow-up from the baseline examination until the date of death or July 31, 2022. Death certificates were obtained from the local health authorities. Cardiovascular disease mortality was defined by the International Classification of Diseases version 10 (ICD-10) codes I00 to I78 [12]. The underlying cause of death was initially verified independently by two internists, and any disagreements were resolved through joint determination with a third internist.

### Statistical analysis

The baseline characteristics of participants are presented as the mean ± standard deviation (SD) for continuous variables with a normal distribution and as frequency (percentage) for classified variables. Group differences for continuous variables were assessed using the t-test. Reliability was evaluated by the intraclass correlation coefficient (ICC). The paired t-test was applied to examine differences between the two measurements.

Cox proportional-hazard models were utilized to explore the association between CCA diameter at baseline and the risk of CVD mortality. Additionally, CCA diameter was categorized into quartiles (Q), where Q1 represented the lowest quartile and Q4 the highest. The specific ranges for CCA diameters were as follows: Q1 (≤ 6.65 mm), Q2 (6.66–7.25 mm), Q3 (7.26–8.05 mm), and Q4 (≥ 8.06 mm) (Table 1). Cardiovascular disease mortality was calculated based on quartiles of carotid artery diameter. Kaplan-Meier curves were employed to compare CVD mortality risk stratified by CCA diameter quartile, followed by a log-rank test.

Furthermore, the analyses were conducted in three models: model 1 was unadjusted; model 2 was adjusted for sex and age; and model 3 was further adjusted for education level, income, heart rate, body mass index, smoking, history of stroke, history of coronary heart disease, atrial fibrillation, systolic blood pressure, diabetes, total cholesterol (TC), triglyceride (TG), high-density lipoprotein cholesterol (HDL-C), and low-density lipoprotein cholesterol (LDL-C). All Cox regression analyses adhered to the proportionality assumption. SPSS version 24.0 (IBM Corporation, Armonk, NY, USA) was employed for data processing and analysis. A two-tailed *P* < 0.05 indicated statistical significance for all analyses, and estimates were expressed as a 95% CI.

## Results

### Population characteristics at baseline

Table 1 presents the baseline characteristics of the study population and carotid diameter quartiles. The study included 5668 participants, comprising 2218 males (39.1%) and 3450 females (60.9%). The mean age of the population was 58.9 ± 10.1 years. Approximately 57.4% of participants had an education level of primary school or lower, and 36.0% reported a household income of less than 5000 yuan (approximately $700) per year. Among the participants, 25.7% were current smokers, 57.3% had hypertension, and 12.6% had diabetes.

### Characteristic of CCA diameter at baseline

The average CCA diameter by sex and age is summarized in Table 2. The average CCA diameter (mm) was 7.30 ± 0.99, exhibiting a significant increase with age. The diameter ranged from 6.65 ± 0.71 in the 40–49 age group to 7.99 ± 1.04 in the group aged 80 years or older. Additionally, the CCA diameter in males was significantly larger than that in females (7.51 ± 1.03 mm vs. 7.16 ± 0.94 mm; *P* < 0.001). Among males, the CCA diameter increased from 6.86 ± 0.76 mm in the 40–49 age group to 8.06 ± 1.04 mm in those aged ≥ 80 years. Among females, the CCA diameter increased from 6.56 ± 0.67 mm in the 40–49 age group to 7.94 ± 1.05 mm in those aged ≥ 80 years.

**Table 2:** The average CCA (mm) by sex and age

**Age, years**	**Men**	**Women**	**Total**	***P* value**
40–49	6.86 ± 0.76	6.56 ± 0.67	6.65 ± 0.71	< 0.001
50–59	7.26 ± 0.87	6.90 ± 0.79	7.03 ± 0.84	< 0.001
60–69	7.73 ± 1.03	7.57 ± 0.88	7.64 ± 0.95	< 0.001
70–79	8.09 ± 1.04	7.87 ± 0.89	7.97 ± 0.97	0.002
≥ 80	8.06 ± 1.04	7.94 ± 1.05	7.99 ± 1.04	0.470
Total	7.51 ± 1.03	7.16 ± 0.94	7.30 ± 0.99	< 0.001

Continuous variables are expressed as mean ± standard deviation (SD)

*CCA* Common carotid artery

The ICC between the initial and subsequent CCA measurements, conducted with a 1-week interval for 50 participants, was found to be 0.92 (*P* < 0.001). This excellent ICC value indicates high reliability. The mean difference between the measurements was 0.062 ± 0.25 mm (*P* = 0.085).

### Association between CCA diameter and cardiovascular disease

During the median follow-up of 4.48 years, a total of 185 participant died of cardiovascular disease. The cumulative CVD mortality rate increased with the rise in CCA diameter, reaching 7.4% in the highest quartile (Q4) compared to 0.9% in the lowest quartile (Q1).

Cox proportional-hazard models were employed to investigate the relationship between CCA diameter and CVD mortality. Individuals in the highest quartile of carotid diameter (diameter ≥ 8.06 mm), compared with those in the lowest quartile (diameter ≤ 6.65 mm), had a substantially higher risk of developing adverse outcomes related to cardiovascular disease (model1: HR: 8.75; 95% CI: 4.98–15.40). In the fully adjusted model, the risk of CVD mortality in the highest quartile (Q4) was 2.29 times higher (95% CI: 1.24, 4.22) than that in the lowest quartile (Q1). Additionally, a per 1 SD increase in the diameter of the common carotid artery (per standard deviation) was associated with a 36% increased risk of CVD mortality (HR: 1.36; 95% CI: 1.18–1.57) (Table 3).

**Table 3:** Association between baseline CCA and the risk of CVD mortality

**CCA quartiles**	**CVD mortality/participants**	**Model 1**		**Model 2**		**Model 3**	
		**HR (95% CI)**	***P* value**	**HR (95% CI)**	***P* value**	**HR (95% CI)**	***P* value**
Q1: <= 6.65 mm	14/1609	Reference		Reference		Reference	
Q2: 6.66–7.25 mm	29/1423	2.33 (1.23, 4.41)	0.009	1.54 (0.81, 2.92)	0.188	1.50 (0.78, 2.91)	0.226
Q3: 7.26–8.05 mm	56/1476	4.38 (2.44, 7.86)	< 0.001	1.70 (0.93, 3.09)	0.083	1.57 (0.84, 2.93)	0.160
Q4: >= 8.06 mm	86/1160	8.75 (4.98, 15.40)	< 0.001	2.59 (1.45, 4.64)	0.001	2.29 (1.24, 4.22)	< 0.01
Per SD increase	185/5668	1.95 (1.73, 2.19)	< 0.001	1.40 (1.22, 1.61)	< 0.001	1.36 (1.18, 1.57)	< 0.001

*CCA* Common carotid artery, *CVD* Cardiovascular disease, *Q* Quartile, *HR* Hazard ratio, *CI* Confidence interval

Model 1: unadjusted, Model 2: adjusted for sex and age, Model 3: further adjusted for education level, income, heart rate, body mass index, smoking, history of stroke, history of coronary heart disease, atrial fibrillation, systolic blood pressure, diabetes, total cholesterol, triglyceride, high-density lipoprotein cholesterol, low-density lipoprotein cholesterol

**Fig. 2:**
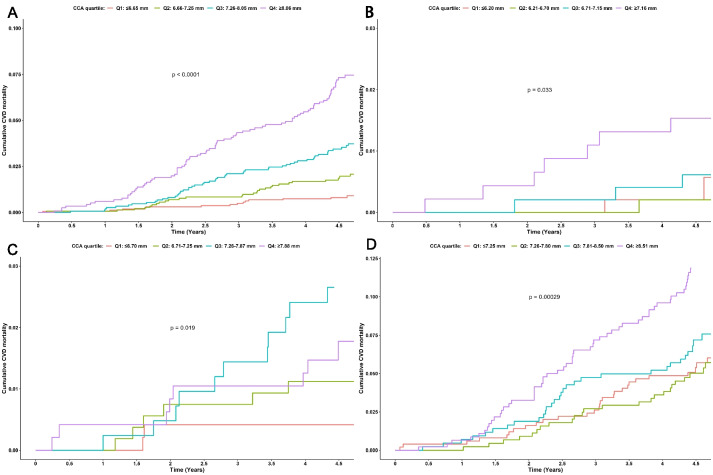
Kaplan-Meier curves for the cumulative risk of cardiovascular disease mortality for overall (**A**), age tertile 1 (≤ 53 years) (**B**), age tertile 2 (54–63 years) (**C**), age tertile 3 (≥ 64 years) (**D**), respectively

The results of subgroup analysis revealed a higher number of CVD deaths in age tertile 3 (142/1837) compared to tertile 1 (14/1915) and tertile 2 (29/1916). A per SD increase was associated with a 42% increased risk of CVD mortality in participants aged ≥ 64 years (HR: 1.42; 95% CI: 1.21, 1.66), indicating an increased risk in the older age group. The association between baseline CCA and the risk of CVD mortality was statistically significant only in the older age group (age tertile 3) (Table 4). The Kaplan-Meier curves depicted that the risk of cardiovascular disease mortality in the highest quartile (Q4) was the highest among all quartiles, and this pattern persisted when stratified by age tertiles (Fig. 2).

**Table 4:** Association between CCA quartiles and CVD mortality risk, stratified by age tertiles

**CCA quartiles**	**CVD mortality/participants**	**Model 1**		**Model 2**		**Model 3**	
		**HR (95% CI)**	***P* value**	**HR (95% CI)**	***P* value**	**HR (95% CI)**	***P* value**
**Age tertile 1:** ≤ **53 y**							
Q1: <= 6.20 mm	2/484	Reference		Reference		Reference	
Q2: 6.21–6.70 mm	1/487	0.49 (0.04, 5.39)	0.558	0.43 (0.04, 4.78)	0.494	0.45 (0.04, 5.31)	0.527
Q3: 6.71–7.15 mm	3/487	1.46 (0.24, 8.73)	0.679	1.13 (0.19, 6.90)	0.893	0.98 (0.15, 6.26)	0.979
Q4: >= 7.16 mm	8/457	4.08 (0.87, 19.25)	0.075	2.81 (0.57, 13.71)	0.202	1.69 (0.29, 10.0)	0.562
Per SD increase	14/1915	1.75 (1.23, 2.49)	< 0.01	1.64 (1.10, 2.46)	< 0.001	1.54 (0.91, 2.63)	0.11
**Age tertile 2: 54–63 y**							
Q1: <= 6.70 mm	2/483	Reference		Reference		Reference	
Q2: 6.71–7.25 mm	6/536	2.71 (0.55, 13.41)	0.223	2.29 (0.46, 11.39)	0.31	1.57 (0.31, 8.08)	0.588
Q3: 7.26–7.87 mm	12/418	6.95 (1.56, 31.05)	0.011	5.48 (1.22, 24.62)	0.026	4.61 (0.98, 21.74)	0.053
Q4: >= 7.88 mm	9/479	4.53 (0.98, 20.96)	0.053	2.97 (0.63, 13.99)	0.168	1.51 (0.29, 7.73)	0.624
Per SD increase	29/1916	1.53 (1.10, 2.13)	0.012	1.32 (0.94, 1.87)	0.112	1.18 (0.80, 1.75)	0.41
**Age tertile 3:** ≥ **64 y**							
Q1: <= 7.25 mm	29/500	Reference		Reference		Reference	
Q2: 7.26–7.80 mm	26/448	0.99 (0.58, 1.68)	0.969	0.85 (0.50, 1.45)	0.55	0.85 (0.49, 1.47)	0.558
Q3: 7.81–8.50 mm	30/428	1.23 (0.74, 2.05)	0.425	1.10 (0.66, 1.83)	0.724	1.05 (0.62, 1.78)	0.853
Q4: >= 8.51 mm	57/461	2.18 (1.40, 3.41)	0.001	1.88 (1.20, 2.95)	0.006	1.92 (1.19, 3.08)	0.007
Per SD increase	142/1837	1.44 (1.24, 1.67)	< 0.001	1.39 (1.20, 1.62)	< 0.001	1.42 (1.21, 1.66)	< 0.001

*CCA* Common carotid artery, *CVD* Cardiovascular disease, *Q* Quartile, *HR* Hazard ratio, *CI* Confidence interval

Model 1: unadjusted, Model 2: adjusted for sex and age, Model 3: further adjusted for education level, income, heart rate, body mass index, smoking, history of stroke, history of coronary heart disease, atrial fibrillation, systolic blood pressure, diabetes, total cholesterol, triglyceride, high-density lipoprotein cholesterol, low-density lipoprotein cholesterol

## Discussion

In this cohort study, we identified a significant relationship between the diameter of the CCA and CVD mortality in a population from northeast China. Our findings demonstrated that enlargement of CCA diameter was associated with an increased risk of death from cardiovascular disease. This suggests that CCA diameter serves as a crucial predictive factor for the risk of CVD mortality, surpassing the predictive capacity of traditional clinical risk factors. Therefore, the assessment of CCA diameter provides possible independent and incremental value in predicting the prognosis of cardiovascular disease.

Cardiovascular diseases encompass conditions such as coronary heart disease and stroke [13]. A prior study indicated that an enlarged CCA diameter was linked to a higher prevalence of cardiovascular events [11]. Moreover, our previous research demonstrated a positive association between CCA diameter and the prevalence of stroke [14, 15]. These findings align with the results of the present study. However, the association between CCA diameter and CVD mortality was not explored in those studies, leaving the prognostic value of CCA for CVD unclear. Additionally, a retrospective cohort study conducted in Taiwan, China, reported an independent association between reduced CCA diameter and CVD mortality [16]. This finding contrasts with our results. However, the retrospective nature of their study may introduce classification errors and data inaccuracies, making our results more reliable.

Several other studies have also suggested that an enlarged CCA increases the risk of CVD mortality. A case-control study showed that in postmenopausal women, the diameter of the CCA was larger in subjects with atherosclerosis than in those without [17]. CCA enlargement might serve as compensation for atherosclerotic lesions. As plaque volume increased, the carotid segment expanded, and the interplay between these processes compensated for arterial stenosis in the early stages of the disease [18]. Atherosclerosis is considered a primary cause of cardiovascular disease [19], and this finding is consistent with ours, confirming that CCA enlargement is associated with CVD. Another study demonstrated that carotid enlargement is a valid predictor of adverse cardiovascular events [20]. Therefore, carotid diameter contributes to identifying individuals at high cardiovascular risk and provides an effective means to monitor the prognosis of cardiovascular disease.

Nonetheless, the findings of the present study suggest a departure from a linear association between CCA diameter and CVD mortality in individuals under 64 years of age. The correlation between CCA diameter and CVD mortality is intricately tied to age, with distinct patterns observed in the younger group. Within the younger cohort, the risk of CVD mortality appears comparatively low, possibly attributed to a more favorable physiological state. Furthermore, the progression and eventual occurrence of CVD-related mortality typically unfold gradually over an extended period. The limited number of CVD mortality events within this subgroup has, however, posed a challenge to detecting a statistically significant association. To arrive at more definitive conclusions, it is imperative to embark on further investigations incorporating a longer-term follow-up and larger sample sizes. This approach will provide a more comprehensive understanding of the intricate interplay between CCA diameter and CVD mortality, particularly in the context of age-related dynamics.

Cardiovascular disease stands as one of the leading causes of death and disability-adjusted life years worldwide. The rise in CVD deaths is fueled by population growth and aging, particularly in South and East Asian countries with large and expanding populations [3]. Consequently, there is a need for more research on cardiovascular disease in Asia. Our study builds upon previous research [21, 22]. Clinicians can utilize the findings of these studies to assess the degree of CCA enlargement, enabling them to grade the severity of the disease and administer appropriate treatment. Thus, the present study holds significant value for monitoring and preventing adverse cardiovascular outcomes in the general population in northeast China.

Nevertheless, our study has certain limitations that need consideration. First, we relied on a single baseline measurement of covariates, and some of these variables may have changed over time, potentially introducing bias toward null values. Second, many previous studies have explored the relationship between IMT and cardiovascular events, but the results vary due to different measurement methods, follow-up time, and populations [5–7, 20]. Therefore, this study mainly focused on the change of the CCA diameter, we did not include IMT in the present study, however, we intend to investigate IMT and other parameters after longer follow-up in the future. Third, our study population was drawn from rural areas in northeast China; hence, further research in diverse regions is essential to enhance our understanding of the relationship between CCA diameter and cardiovascular disease mortality.

## Conclusion

In conclusion, our study highlights a substantial association between CCA diameter and CVD mortality in a population from northeast China. Specifically, enlargement of CCA diameter appears to be linked to an increased risk of death from cardiovascular disease, especially in the elderly individuals. However, the relationship between CCA diameter and CVD mortality does not exhibit an obvious linear pattern in the younger population, a longer-term follow-up and larger sample sizes are required. Moreover, our findings propose that CCA diameter serves as a possible predictor of CVD prognosis and offers potential incremental value in refining the risk stratification of cardiovascular disease. This information could be crucial in efforts to mitigate the burden of cardiovascular disease in China.

## Data Availability

The datasets generated for and analyzed in the study are not publicly available due to China Medical University’s privacy policy but are available from the corresponding author on reasonable request.

## Abbreviations

CCA: Common carotid artery
CVD: Cardiovascular disease
HR: Hazard ratio
CI: Confidence interval
IMT: Intima-media thickness
CHD: Coronary heart disease
SBP: Systolic blood pressure
DBP: Diastolic blood pressure
TC: Total cholesterol
TG: Triglyceride
HDL-C: High-density lipoprotein cholesterol
LDL-C: Low-density lipoprotein cholesterol
